# Using Polynomials to Simplify Fixed Pattern Noise and Photometric Correction of Logarithmic CMOS Image Sensors

**DOI:** 10.3390/s151026331

**Published:** 2015-10-16

**Authors:** Jing Li, Alireza Mahmoodi, Dileepan Joseph

**Affiliations:** Innovation Centre for Engineering, University of Alberta, 9211 116 Street NW, Edmonton, AB T6G 1H9, Canada; E-Mails: jl11@ualberta.ca (J.L.); mahmoodi@ualberta.ca (A.M.)

**Keywords:** logarithmic CMOS image sensor, fixed pattern noise, photometry, Taylor series, polynomial regression, spline interpolation, fixed-point arithmetic, look-up table

## Abstract

An important class of complementary metal-oxide-semiconductor (CMOS) image sensors are those where pixel responses are monotonic nonlinear functions of light stimuli. This class includes various logarithmic architectures, which are easily capable of wide dynamic range imaging, at video rates, but which are vulnerable to image quality issues. To minimize fixed pattern noise (FPN) and maximize photometric accuracy, pixel responses must be calibrated and corrected due to mismatch and process variation during fabrication. Unlike literature approaches, which employ circuit-based models of varying complexity, this paper introduces a novel approach based on low-degree polynomials. Although each pixel may have a highly nonlinear response, an approximately-linear FPN calibration is possible by exploiting the monotonic nature of imaging. Moreover, FPN correction requires only arithmetic, and an optimal fixed-point implementation is readily derived, subject to a user-specified number of bits per pixel. Using a monotonic spline, involving cubic polynomials, photometric calibration is also possible without a circuit-based model, and fixed-point photometric correction requires only a look-up table. The approach is experimentally validated with a logarithmic CMOS image sensor and is compared to a leading approach from the literature. The novel approach proves effective and efficient.

## 1. Introduction

CMOS image sensors suffer from mismatch and process variation during fabrication. Despite identical circuit designs, each pixel on a die responds differently to light stimulus because of device mismatch. This causes FPN in images of uniform or non-uniform stimuli. Moreover, process variation from wafer to wafer contributes further uncertainty to pixel responses, which complicates photometry, *i.e.*, the estimation of true light stimuli from image data. Although relevant to linear imagers, these problems are worse with nonlinear imagers because of their increased circuit complexity.

Nonlinear CMOS image sensors, such as logarithmic (log) and linear-logarithmic (lin-log) designs, achieve dynamic ranges (DRs) of over 120dB easily at video rates, 40–60dB wider than that of charge coupled device (CCD) and linear CMOS image sensors [1,2]. However, our work aside [3], nonlinear imagers suffer, in the log region, from low (below 40dB) peak signal-to-noise-and-distortion ratios (PSNDRs), a measure of image quality that depends on temporal noise and residual FPN. Unfortunately, correlated double sampling (CDS), a simple FPN correction method that is effective with linear CMOS image sensors, does not benefit nonlinear ones, in the log region, to the same extent.

A variety of *analog* approaches, including CDS, have been investigated to correct offset FPN in nonlinear imagers [4,5,6,7,8,9]. Their advantage is that calibration is not required. However, nonlinear imagers are subject, especially in the log region, to higher-order FPN [10,11], such as but not limited to gain FPN, which analog approaches do not correct. Meanwhile, using *digital* FPN (and photometric) correction, as well as a novel architecture, we were first to demonstrate a high PSNDR (45dB)—comparable to that of CCD and linear CMOS image sensors—with a log CMOS image sensor [3].

The calibration and correction we used involved nonlinear regression on a circuit-based model from Joseph and Collins [10]. Although the model and its explanation of FPN have been widely accepted, both for log imagers and the log region of lin-log imagers, researchers have developed a variety of simplifications both to the model itself and to parameter estimation [12,13,14]. Their objectives have been to simplify calibration and correction, while achieving sufficient accuracy. Outperforming the accuracy of the original method, over a wide DR, is practically impossible. With some reasonable assumptions on camera noise, the original method is equivalent to maximum-likelihood estimation [10].

This paper proposes a novel approach for the calibration and correction of nonlinear imagers in general, and for a log imager in particular. The objective of the novel approach is simplified calibration and correction, while achieving sufficient accuracy over a wide DR. Unlike the literature, no circuit-based model is required or used. The approach depends primarily on the monotonic property of pixel responses, a property shared by linear, log, and lin-log imagers. Unlike the literature, with the notable exception of Hoefflinger [13], we also provide a fixed-point implementation of our method, and we prove, unlike Hoefflinger, that the implementation satisfies an optimality criterion.

Section 2 presents our materials and methods, which are, respectively, a log CMOS image sensor, with low temporal noise, and a new approach for FPN and photometric calibration and correction. Section 3 gives a conceptual overview, a mathematical formulation, and important refinements for an optimal fixed-point implementation of the proposed FPN correction. Fixed-point photometric correction is also explained. Section 4 presents experimental results, discussed with respect to the literature, to validate the new methods and implementation. Finally, Section 5 summarizes our contributions.

## 2. Materials and Methods

This section proposes new methods for the calibration and correction of non-idealities in image sensors due to mismatch and process variation. Instead of analytical methods, whereby circuit-based models are used to derive specific calibrations and corrections, numerical methods are applied to general image sensors, modeled using low-degree polynomials. The proposed approach results in efficient methods applicable to a variety of imagers, linear and nonlinear. Nevertheless, a specific log imager and its measured data, our materials, is first presented to put subsequent ideas in context.

### 2.1. Image Sensor

Previously, in this journal, we presented a CMOS digital pixel sensor (DPS) array with a log delta-sigma (△∑) architecture [3]. This image sensor employs a classic log sensor and a novel first-order △∑ analog-to-digital converter (ADC), including decimator, in each pixel. Detailed specifications were provided and the image sensor was compared to a wide variety of other image sensors. For a different purpose than in the prior publication, we revisit the same image sensor and experimental setup.

Details of the experimental setup are given in our prior publication [3]. As previously reported, “The measured data comprises mn[o] pixel responses, $y_{ijk}$, where: *i* indexes luminance stimuli, $x_{i}$, with 1≤i≤m; *j* indexes pixels of the image sensor, with 1≤j≤n; and *k* indexes consecutive frames, with 1≤k≤[o]... [The] index dimensions *m*, *n* and [*o*] are 22, [48×64] and 49, respectively.” For this work, we partition the measured data, comprising mo images (*n* pixels each) of uniform stimuli $x_{i}$, into *m* time-averaged *calibration* images, ${\bar{y}}_{ij}$, and *m* single-frame *additional* images, $y_{ij}$, as follows:

(1) $$\begin{array}{cc} {\bar{y}}_{ij} & =\frac{1}{o-1}\sum_{k=1}^{o-1}y_{ijk} \end{array}$$

(2) $$\begin{array}{cc} y_{ij} & =y_{ijo} \end{array}$$

**Figure 1:**
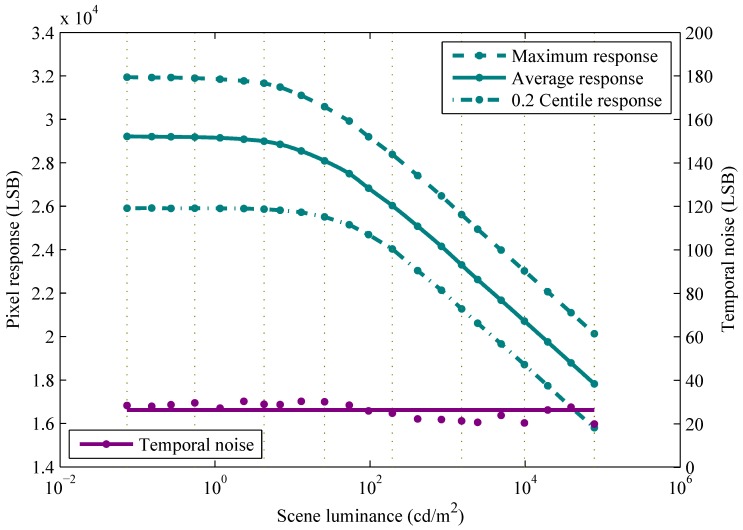
Statistics of a logarithmic (log) imager. The maximum response, average response, minimum response excluding outliers (<0.2% of pixels), and root mean square (RMS) temporal noise per luminance, of a 48×64 pixel array, are shown versus scene luminance. The overall RMS temporal noise is also shown. LSB stands for least significant bit.

Using the calibration data, Figure 1 shows the average response, ${\bar{y}}_{i}$, and the root mean square (RMS) temporal noise per luminance, ${\hat{\sigma }}_{i}^{n}$, of the log CMOS image sensor. These are calculated as follows:

(3) $$\begin{array}{cc} {\bar{y}}_{i} & =\frac{1}{n}\sum_{j=1}^{n}{\bar{y}}_{ij} \end{array}$$

(4) $$\begin{array}{cc} {\hat{\sigma }}_{i}^{n} & =\sqrt{\frac{1}{n(o-2)}\sum_{j=1}^{n}\sum_{k=1}^{o-1}{\left(r_{ijk}^{n}\right)}^{2}} \end{array}$$

where

(5) $$\begin{array}{cc} r_{ijk}^{n} & =y_{ijk}-{\bar{y}}_{ij} \end{array}$$

The overall RMS temporal noise, also shown in Figure 1, is calculated as follows:

(6) $$\begin{array}{cc} {\hat{\sigma }}^{n} & =\sqrt{\frac{1}{mn(o-2)}\sum_{i=1}^{m}\sum_{j=1}^{n}\sum_{k=1}^{o-1}{\left(r_{ijk}^{n}\right)}^{2}} \end{array}$$

As shown in Figure 1, the range of pixel responses, at each uniform luminance, is quite large, indicative of substantial FPN. As disclosed previously [3], a small number of pixel responses (*i.e.*, six) were outliers because of in-pixel ADC issues. While excluded from the range shown in Figure 1, they are not excluded in all other results, serving to demonstrate the robustness of our approach.

Over a wide DR, the average response, ${\bar{y}}_{i}$, is a monotonic nonlinear function of scene luminance, $x_{i}$. At present, no theory has been published to explain the temporal noise of log pixels with in-pixel △∑ ADCs. Experimentally, the RMS temporal noise per luminance, ${\hat{\sigma }}_{i}^{n}$, proves relatively independent of luminance. Moreover, the temporal noise residuals, $r_{ijk}^{n}$, approximately follow a normal distribution, and their RMS values are also relatively independent of pixel index, *j*, and frame index, *k*.

High peak signal-to-noise ratios (PSNRs), which are independent of FPN correction, are important because they limit PSNDRs. With our log imager [3], over 95% of pixels have a PSNR of at least 40dB, and the median PSNR is about 46dB. Storm *et al.* [12] report a PSNR of about 32dB, in the log region, for their lin-log imager, and Hoefflinger [13] reports a PSNR of about 35dB for his log imager.

As mentioned in Section 1, we aim to simplify correction, while achieving sufficient accuracy over a wide DR. One degree of complexity, normal for a log imager, is a reduced sensitivity (slope) in the dimmer 60dB of the over 120dB of tested DR. Otim *et al.* [14], for example, propose a simplified correction that would only apply to the brighter 60dB. Another degree of complexity is that, because our PSNR is relatively high, our FPN correction has to be more accurate, for residual FPN to be on the same order as temporal noise. Fortunately, our proposed approach handles these complexities.

### 2.2. Polynomial Regression

As with a literature method [10], calibration uses *m* images of *uniform* stimuli $x_{i}$, where 1≤i≤m. For an image sensor with *n* pixels, the *actual* responses ${\bar{y}}_{ij}$, where 1≤j≤n, are modeled thusly:

(7) $$\begin{array}{cc} {\bar{y}}_{ij} & =f_{j}\left(x_{i}\right)+\epsilon_{ij} \end{array}$$

where $f_{j}$ are monotonic functions that vary with pixel index *j* due to device mismatch.

Residual errors $\epsilon_{ij}$, in Equation (7), encapsulate temporal noise, including quantization noise, and residual FPN due to imperfect modeling. The advantage of using time-averaged actual responses for calibration, indicated by a bar on $y_{ij}$, is that temporal noise power is reduced, allowing methods to focus on residual FPN. Errors are assumed to behave as independent and identically-distributed random variables, which follow a zero-mean normal distribution. If standard deviations of residual errors were to depend on stimuli, a weighting scheme may be used to factor out such dependence.

FPN occurs, unfortunately, because $f_{j}$ varies randomly from pixel to pixel due to device mismatch. With colour image sensors, $f_{j}$ varies also by design. In that case, the theory presented here applies to red, green, and blue pixels when treated separately. Average responses ${\bar{y}}_{i}$ of all pixels, as follows, may be considered the *ideal* responses $F(x_{i})$ of the image sensor to the same uniform stimuli:

(8) $$\begin{array}{cc} {\bar{y}}_{i}=\frac{1}{n}\sum_{j=1}^{n}{\bar{y}}_{ij} & \approx \frac{1}{n}\sum_{j=1}^{n}f_{j}\left(x_{i}\right)\equiv F\left(x_{i}\right) \end{array}$$

Because of their zero-mean normal distribution, residual errors are effectively filtered out of the ideal responses, in Equation (8), when *n* is large enough, a good assumption for an image sensor of many pixels. Moreover, if each $f_{j}$ is monotonic with respect to $x_{i}$, also a good assumption for an image sensor, then so is *F*. Furthermore, if *F* is monotonic then its inverse $F^{-1}$ exists and the following holds:

(9) $$\begin{array}{cc} x_{i} & =F^{-1}\left({\bar{y}}_{i}\right) \end{array}$$

Using Equations (7) and (9), actual responses ${\bar{y}}_{ij}$ may be written in terms of ideal responses ${\bar{y}}_{i}$:

(10) $$\begin{array}{c} {\bar{y}}_{ij}=f_{j}\left(F^{-1}\left({\bar{y}}_{i}\right)\right)+\epsilon_{ij} \end{array}$$

Although $f_{j}$ and $F^{-1}$ are functions that may be quite nonlinear, their composition in Equation (10) is expected to be less so because of an approximate inverse relationship. Notably, these functions would be exact inverses if there were no mismatch variation. Using Taylor’s theorem, the composite functions are replaced by ${\bar{y}}_{i}$ perturbed by degree-*p* polynomials, with low *p* expected to suffice, as follows:

(11) $$\begin{array}{c} {\bar{y}}_{ij}={\bar{y}}_{i}+a_{j0}+a_{j1}{\bar{y}}_{i}+\ldots a_{jp}{\bar{y}}_{i}^{p}+\epsilon_{ij} \end{array}$$

where $a_{jk}$ are per-pixel parameters, with 0≤k≤p, and residual errors $\epsilon_{ij}$ absorb truncation errors when *p* is high enough. Given sufficient calibration data, maximum likelihood (ML) estimates ${\hat{a}}_{jk}$ of the p+1 parameters per pixel may be easily computed using the ordinary least squares method.

The above is called the polynomial regression (PR) method for FPN *calibration*. For FPN *correction*, consider the actual responses $y_{j}$ to *arbitrary* (*i.e.*, uniform or non-uniform) stimuli $x_{j}$:

(12) $$\begin{array}{cc} y_{j} & =f_{j}\left(x_{j}\right)+\epsilon_{j} \end{array}$$

Using the same approach as above, it is straightforward to show that the ML estimates ${\hat{y}}_{j}$ of the ideal responses to the arbitrary stimuli are given by roots of *n* degree-*p* polynomial equations, as follows:

(13) $$\begin{array}{cc} y_{j} & ={\hat{y}}_{j}+a_{j0}+a_{j1}{\hat{y}}_{j}+\ldots a_{jp}{\hat{y}}_{j}^{p} \end{array}$$

where the ML estimates ${\hat{a}}_{jk}$, obtained by calibration, are employed for the correction.

With the PR method, calibration is straightforward—it is equivalent to polynomial regression—but correction is relatively difficult—it is equivalent to polynomial root finding. For p≥2, correction requires more than just arithmetic, unlike calibration. However, what matters is for correction to be simple, preferably using a low-power approach, because it has to be done repeatedly in real time, unlike calibration. The PR method above provides a useful foundation for such a method below.

### 2.3. Inverse Polynomial Regression

For efficient video processing in real time, FPN correction should be computed using only arithmetic. Toward this end, monotonic functions in Equation (10) are first inverted to write ${\bar{y}}_{i}$ in terms of ${\bar{y}}_{ij}$:

(14) $$\begin{array}{cc} {\bar{y}}_{i} & =F\left(f_{j}^{-1}\left({\bar{y}}_{ij}-\epsilon_{ij}\right)\right)\approx F\left(f_{j}^{-1}\left({\bar{y}}_{ij}\right)\right)+\epsilon_{ij}^{\prime } \end{array}$$

where

(15) $$\begin{array}{c} \epsilon_{ij}^{\prime }=-\frac{d{\bar{y}}_{i}}{d{\bar{y}}_{ij}}\epsilon_{ij} \end{array}$$

As with the PR method, composite functions in Equation (14) are replaced with Taylor polynomials:

(16) $$\begin{array}{cc} {\bar{y}}_{i} & ={\bar{y}}_{ij}+b_{j0}+b_{j1}{\bar{y}}_{ij}+\ldots b_{jq}{\bar{y}}_{ij}^{q}+\epsilon_{ij}^{\prime } \end{array}$$

where $b_{jk}$, with 0≤k≤q, are per-pixel parameters. Although residual errors $\epsilon_{ij}^{\prime }$ independently follow zero-mean normal distributions, they are not identically distributed due to Equation (15). The weighted least squares method is therefore used to obtain ML estimates ${\hat{b}}_{jk}$ of the q+1 parameters per pixel. Based on the PR method, where *p* is taken to equal *q*, weights $w_{ij}$ are estimated as follows:

(17) $$\begin{array}{cc} {\hat{w}}_{ij} & =1+{\hat{a}}_{j1}+2{\hat{a}}_{j2}{\bar{y}}_{i}+\ldots p{\hat{a}}_{jp}{\bar{y}}_{i}^{p-1}\approx \frac{d{\bar{y}}_{ij}}{d{\bar{y}}_{i}} \end{array}$$

The above is called the inverse polynomial regression (IPR) method for FPN *calibration*. For FPN *correction*, consider again Equation (12), which gives the actual responses $y_{j}$ to arbitrary stimuli $x_{j}$. It is straightforward to show, via the above approach, that the ML estimates ${\hat{y}}_{j}$ of ideal responses to the arbitrary stimuli are given by *n* polynomials, computable without exponents, as follows:

(18) $$\begin{array}{cc} {\hat{y}}_{j} & =y_{j}+b_{j0}+b_{j1}y_{j}+\ldots b_{jq}y_{j}^{q} \end{array}$$

(19) $$\begin{array}{cc} & =y_{j}+b_{j0}+y_{j}\left(b_{j1}+\ldots y_{j}\left(b_{jq}\right)\right) \end{array}$$

where the ML estimates ${\hat{b}}_{jk}$, obtained by calibration, are employed for the correction.

Although IPR calibration is more complex than PR calibration, it can still be done using only arithmetic. Moreover, the extra complexity is insignificant because calibration, unlike correction, is done once and need not be embedded with an image sensor. On the other hand, IPR correction is much simpler than PR correction. It requires a small number of additions and multiplications per pixel, a substantial simplification. Arithmetic operations are especially efficient for real-time processing.

### 2.4. Inverse Spline Interpolation

The IPR method corrects FPN in actual responses $y_{j}$ to arbitrary stimuli $x_{j}$ by mapping the former to ideal responses ${\hat{y}}_{j}$. It is a *relative* calibration, addressing intra-die mismatch variation. To address intra-wafer (die-to-die) and inter-wafer process variation, an *absolute* calibration is also required. The focus of this paper is on log CMOS image sensors, especially for wide DR imaging. Ideal responses ${\hat{y}}_{j}$ are therefore calibrated absolutely with respect to estimated stimuli $\ln{\hat{x}}_{j}$ on a log scale, not ${\hat{x}}_{j}$ on a linear scale. That way, fewer bits are needed for satisfactory encoding of wide DR responses.

Data collected for the FPN calibration, *i.e.*, the average responses ${\bar{y}}_{i}$ to the *m* uniform stimuli $x_{i}$, is also used to perform the photometric calibration. Given Equation (9), the following holds:

(20) $$\begin{array}{cc} \ln x_{i} & =\ln F^{-1}\left({\bar{y}}_{i}\right) \end{array}$$

where $\ln F^{-1}$ is monotonic. Instead of using circuit analysis of the nonlinear pixel to model the relationship, a cubic Hermite spline *S* is constructed to interpolate the *m* data points monotonically:

(21) $$\begin{array}{cc} \ln x_{i} & =S({\bar{y}}_{i}) \end{array}$$

where

(22) $$\begin{array}{cc} S(y) & =\left\{\begin{array}{cc} S_{1}(y), & y\leq {\bar{y}}_{2}\\ S_{2}(y), & {\bar{y}}_{2}<y\leq {\bar{y}}_{3}\\ \vdots & \vdots \\ S_{m-1}(y), & {\bar{y}}_{m-1}<y \end{array}\right. \end{array}$$

The cubic polynomials $S_{i}$, in Equation (22), may be computed using only arithmetic, as follows:

(23) $$S_{i}\left(y\right)=c_{i0}+\left(y-{\bar{y}}_{i}\right)\left(c_{i1}+\left(y-{\bar{y}}_{i}\right)\left(c_{i2}+\left(y-{\bar{y}}_{i}\right)\left(c_{i3}\right)\right)\right)$$

where the 4(m-1) parameters $c_{ik}$, with 1≤i≤m-1 and 0≤k≤3, are calculated once offline, e.g., using pchip in Matlab, during photometric calibration. The above arithmetic is employed repeatedly, in real time, for the photometric correction of ideal responses ${\hat{y}}_{j}$ to estimated stimuli $\ln{\hat{x}}_{j}$:

(24) $$\begin{array}{cc} \ln{\hat{x}}_{j} & =S({\hat{y}}_{j}) \end{array}$$

This approach is called the inverse spline interpolation (ISI) method because it models an inverse function using spline interpolation. Calibration is straightforward, requiring a well known algorithm for spline interpolation, and correction is efficient, requiring only selection and arithmetic operations.

## 3. Fixed-Point Implementation

The previous section introduced methods for the calibration and correction of mismatch and process variation in CMOS image sensors, particularly log imagers. Unlike calibration, correction must be done efficiently in real time to be suitable for low-power video applications, an important end use of CMOS image sensors. Thus, an optimized fixed-point (integer) implementation is presented here.

**Figure 2:**
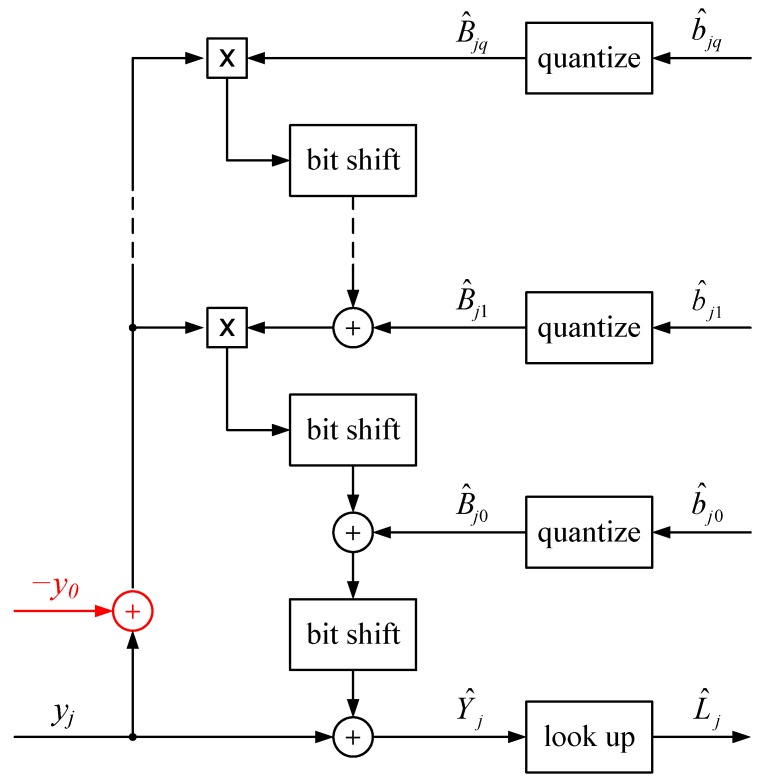
Fixed-point implementation of correction. Quantization and bit shifting introduce static and dynamic round-off errors, respectively, to the fixed pattern noise (FPN) correction. Their impact is minimized, in the fixed-point implementation, subject to a total wordlength limit per pixel. A look-up table (LUT) suffices for the photometric correction.

Static quantization and dynamic bit shifting are used to implement the FPN, *i.e.*, IPR, correction, as shown in Figure 2, whereas a look-up table (LUT) suffices for the photometric, *i.e.*, ISI, correction. Because the output of a fixed-point IPR correction, explained in detail below, is essentially an integer, the LUT is constructed simply by computing, for each possible integer, the result of the ISI correction. Unlike IPR correction, which has per-pixel parameters, ISI correction is the same for each pixel, and so only one LUT is required per imager. Further aspects of the LUT are explained in Section 4.

### 3.1. Conceptual Overview

Actual responses $y_{j}$ to arbitrary stimuli $x_{j}$, where *j* indexes pixels, are assumed to be unsigned integers. These responses are the raw outputs of an image sensor after analog-to-digital conversion. However, the n(q+1) estimated parameters ${\hat{b}}_{jk}$, where *k* indexes parameters, obtained by IPR calibration are floating-point numbers. They may be turned into fixed-point numbers by quantization. As a result, IPR correction, in Equation (19), may be implemented using only fixed-point arithmetic.

Parameter quantization has opposing requirements. Let $t_{k}$, where 0≤k≤q, represent the numbers of bits, *i.e.*, the wordlengths, allocated per parameter, where homogeneity across pixels is employed for simplicity. On the one hand, $t_{k}$ should be large so that the static errors $\Delta {\hat{b}}_{jk}$ added to estimated parameters ${\hat{b}}_{jk}$ do not degrade the IPR correction. On the other hand, the total wordlength $t=\sum_{k=0}^{q}t_{k}$ should be small to reduce the memory and processing required to implement the IPR correction.

The impact of parameter quantization on the residual errors of the IPR calibration is modeled below. This reuses the calibration data, *i.e.*, actual responses ${\bar{y}}_{ij}$ to *m* uniform stimuli $x_{i}$, modeled in Equation (7). Subject to a given total wordlength *t*, e.g., based on an integer number of bytes, the optimal parameter wordlengths $t_{k}$ are computed. For this optimized design, the RMS of residual errors is found for the calibration data. By comparing the RMS residual errors of the floating-point and fixed-point implementations, where the latter is a function of *t*, a suitable total wordlength may be found.

### 3.2. Mathematical Formulation

For either a floating-point or fixed-point implementation of the IPR method, the RMS of residual errors $\epsilon_{ij}$ is directly proportional to the sum square error (SSE) of weighted residuals $w_{ij}\epsilon_{ij}^{\prime }$. With the calibration data, *i.e.*, the actual and ideal responses ${\bar{y}}_{ij}$ and ${\bar{y}}_{i}$, the fixed-point SSE is given by:

(25) $$\begin{array}{cc} {\mathrm{SSE}}^{\mathrm{fixed}} & =\sum_{i=1}^{m}\sum_{j=1}^{n}{\hat{w}}_{ij}^{2}{\left({\bar{y}}_{i}-{\hat{Y}}_{ij}\right)}^{2} \end{array}$$

(26) $$\begin{array}{cc} {\hat{Y}}_{ij} & ={\hat{y}}_{ij}+\Delta {\hat{y}}_{ij} \end{array}$$

(27) $$\begin{array}{cc} {\hat{y}}_{ij} & ={\bar{y}}_{ij}+{\hat{b}}_{j0}+{\bar{y}}_{ij}\left({\hat{b}}_{j1}+\ldots {\bar{y}}_{ij}\left({\hat{b}}_{jq}\right)\right) \end{array}$$

where ${\hat{y}}_{ij}$ and ${\hat{Y}}_{ij}$ are the floating and fixed-point corrections of ${\bar{y}}_{ij}$, respectively, and ${\hat{w}}_{ij}$ are the weights in Equation (17). Correction errors $\Delta {\hat{y}}_{ij}$ are polynomial functions of quantization errors $\Delta {\hat{b}}_{jk}$:

(28) $$\begin{array}{cc} \Delta {\hat{y}}_{ij} & =\Delta {\hat{b}}_{j0}+{\bar{y}}_{ij}\left(\Delta {\hat{b}}_{j1}+\ldots {\bar{y}}_{ij}\left(\Delta {\hat{b}}_{jq}\right)\right) \end{array}$$

Direct optimization of Equation (25) proved too difficult. Instead, assume that the quantization errors $\Delta {\hat{b}}_{jk}$ behave as independent random variables that are uniformly distributed as follows:

(29) $$\begin{array}{cc} |\Delta {\hat{b}}_{jk}| & \leq 0.5e_{k} \end{array}$$

(30) $$\begin{array}{cc} e_{k} & =2^{s_{k}} \end{array}$$

where $e_{k}$ and $s_{k}$ are the quantization step sizes and binary-point positions, respectively. Then, using Equation (28) and the calculus of random variables, the following expectations may be derived:

(31) $$\begin{array}{cc} E\{\Delta {\hat{y}}_{ij}\} & =0 \end{array}$$

(32) $$\begin{array}{cc} E\{\Delta {\hat{y}}_{ij}^{2}\} & =\frac{1}{12}\sum_{k=0}^{q}{\bar{y}}_{ij}^{2k}e_{k}^{2} \end{array}$$

Equation (29) requires that quantization errors $\Delta {\hat{b}}_{jk}$ be bounded by $\pm 0.5\;e_{k}$, which is true only if saturation is avoided. This implies the following conditions on minimum parameter wordlengths $t_{k}$:

(33) $$\begin{array}{cc} t_{k} & =\lceil \log_{2}\left(1+d_{k}/e_{k}\right)\rceil \end{array}$$

(34) $$\begin{array}{cc} d_{k} & =\max_{j}\left\{{\hat{b}}_{jk}\right\}-\min_{j}\left\{{\hat{b}}_{jk}\right\} \end{array}$$

where $d_{k}$ represent the static ranges of the estimated floating-point parameters ${\hat{b}}_{jk}$.

For fixed-point implementation purposes, the only random variables in Equations (25)–(28) are the quantization errors $\Delta {\hat{b}}_{jk}$. Other symbols represent constants obtained via floating-point IPR calibration and correction. Using the calculus of random variables, the expected SSE may be derived:

(35) $$\begin{array}{cc} E\{{\mathrm{SSE}}^{\mathrm{fixed}}\} & ={\mathrm{SSE}}^{\mathrm{float}}+E\left\{\Delta \mathrm{SSE}\right\} \end{array}$$

(36) $$\begin{array}{cc} {\mathrm{SSE}}^{\mathrm{float}} & =\sum_{i=1}^{m}\sum_{j=1}^{n}{\hat{w}}_{ij}^{2}{\left({\bar{y}}_{i}-{\hat{y}}_{ij}\right)}^{2} \end{array}$$

(37) $$\begin{array}{cc} E\{\Delta \mathrm{SSE}\} & =\sum_{i=1}^{m}\sum_{j=1}^{n}{\hat{w}}_{ij}^{2}E\left\{\Delta {\hat{y}}_{ij}^{2}\right\} \end{array}$$

which may be rewritten in terms of quantization step sizes $e_{k}$. Using Equation (32), we obtain:

(38) $$\begin{array}{cc} E\{\Delta \mathrm{SSE}\} & =\sum_{k=0}^{q}c_{k}e_{k}^{2} \end{array}$$

(39) $$\begin{array}{cc} c_{k} & =\frac{1}{12}\sum_{i=1}^{m}\sum_{j=1}^{n}{\left({\hat{w}}_{ij}{\bar{y}}_{ij}^{k}\right)}^{2} \end{array}$$

The expected extra SSE, in Equation (38), may be minimized, subject to total wordlength *t*, with respect to binary-point positions $s_{k}$, via Equation (30), by minimizing the following Lagrangian:

(40) $$\begin{array}{cc} L(s_{k},\lambda ) & =\log_{2}\left(E\left\{\Delta \mathrm{SSE}\right\}\right)+\lambda \left(t-\sum_{k=0}^{q}t_{k}\right) \end{array}$$

where a base-2 logarithm is used for numerical reasons. The Lagrangian is optimized when its gradient is zero. Approximations are required to derive this gradient. Binary-point positions $s_{k}$ and wordlengths $t_{k}$ are treated as reals, although they are integers, and Equation (33) is replaced with the following:

(41) $$\begin{array}{cc} t_{k} & \approx \log_{2}\left(1+d_{k}/e_{k}\right)+0.5 \end{array}$$

where the offset is needed for unbiasedness. The gradient of the Lagrangian is then derived:

(42) $$\begin{array}{cc} \frac{\partial L}{\partial s_{k}} & =\frac{2c_{k}e_{k}^{2}}{E\{\Delta \mathrm{SSE}\}}+\lambda \frac{d_{k}/e_{k}}{1+d_{k}/e_{k}} \end{array}$$

(43) $$\begin{array}{cc} \frac{\partial L}{\partial \lambda } & =t-\sum_{k=0}^{q}t_{k} \end{array}$$

Optimization may be performed using fmincon in Matlab, which chooses an initial *λ* value automatically. Initial $s_{k}$ values are obtained by setting $t_{k}=t/\left(q+1\right)$, solving Equation (41) for $e_{k}$ using $d_{k}$ in Equation (34), and solving Equation (30) for $s_{k}$. Final $s_{k}$ values are rounded to the nearest integers, whereupon integer $t_{k}$ values are computed using Equations (30) and (33). The expected extra SSE in Equation (38) and the expected SSE in Equation (35) are then recomputed.

### 3.3. Important Refinements

A number of details are best explained as refinements to the above formulation. For example, instead of the initially obvious Equation (34), the following $d_{k}$ values are the ones used in the optimization:

(44) $$\begin{array}{cc} d_{k} & =2\max_{j}\left\{\right|{\hat{b}}_{jk}\left|\right\} \end{array}$$

Compared to Equation (34), Equation (44) overstates the ranges of estimated parameters. However, it is unlikely to do so by much, which can be argued via a symmetry analysis of the IPR calibration. On the other hand, Equation (44) simplifies the fixed-point implementation because quantized (${\hat{B}}_{jk}$) and unquantized (${\hat{b}}_{jk}$) parameters are then related only by binary-point shifting and rounding:

(45) $$\begin{array}{cc} {\hat{B}}_{jk} & =\mathrm{round}(2^{-s_{k}}{\hat{b}}_{jk}) \end{array}$$

(46) $$\begin{array}{cc} 2^{s_{k}}{\hat{B}}_{jk} & ={\hat{b}}_{jk}+\Delta {\hat{b}}_{jk} \end{array}$$

With Equation (44), there is no need to quantize, store, and use minimum values of ${\hat{b}}_{jk}$, as with Equation (34). Quantized parameters ${\hat{B}}_{jk}$ are stored, without saturation, using $t_{k}$-bit signed integers. Given actual responses $y_{j}$ to arbitrary stimuli $x_{j}$, fixed-point IPR correction therefore becomes:

(47) $${\hat{Y}}_{j}=y_{j}+2^{s_{0}}\left({\hat{B}}_{j0}+2^{s_{1}-s_{0}}y_{j}\left({\hat{B}}_{j1}+\ldots 2^{s_{q}-s_{q-1}}y_{j}\left({\hat{B}}_{jq}\right)\right)\right)$$

As shown in Figure 2, fixed-point IPR correction involves repeated fixed-point multiplication, bit shifting, and fixed-point addition. Cascading multiplications naively could require the processing of very large words in real time. When *u* and *v*-bit words are multiplied, the result may be a (u+v)-bit word. However, because the IPR method replaces approximately linear functions with Taylor polynomials, most bit shifts will produce insignificant fractional parts. If rounded bit shifting is used, large words are avoided. Moreover, fixed-point arithmetic may be replaced with simple integer arithmetic.

While rounded bit shifting is easily implemented, it could increase the expected extra SSE in Equation (38), which may be considered during optimization. In Equation (47), an addition with ${\hat{B}}_{jk}$ follows each $s_{k+1}-s_{k}$ bit shift. Assuming the shift produces a fractional part, the round-off error may be represented by an independent random variable that is uniformly distributed over a ±0.5LSB range. Instead of adding these random variables to ${\hat{B}}_{jk}$ in Equation (47), they may be scaled by $2^{s_{k}}$ and added to ${\hat{b}}_{jk}$ because of Equation (46). If $s_{0}<0$, the leftmost shift in Equation (47) is also subject to round-off error over a ±0.5LSB range. However, $s_{0}\geq 0$ is likely in an optimal design given that $e_{0}=2^{s_{0}}$ is the precision of ${\hat{b}}_{j0}$, which need not be smaller than 1LSB, the precision of unsigned integers $y_{j}$.

Using the above insights and the calculus of random variables, dynamic round-off errors are modeled by doubling the variances of static round-off errors $\Delta {\hat{b}}_{jk}$ when $s_{k+1}<s_{k}$, assumed to be always true for simplicity. This is captured in the optimization by replacing Equation (39) with the following:

(48) $$\begin{array}{cc} c_{k} & =\frac{\alpha_{k}}{12}\sum_{i=1}^{m}\sum_{j=1}^{n}{\left({\hat{w}}_{ij}{\bar{y}}_{ij}^{k}\right)}^{2} \end{array}$$

where

(49) $$\begin{array}{cc} \alpha_{k} & =\left\{\begin{array}{cc} 2, & 0\leq k\leq q-1\\ 1, & k=q \end{array}\right. \end{array}$$

Finally, due to Equations (33) and (44), parameter wordlengths are proportional to their ranges, all else being constant. These ranges may be significantly reduced by subtracting a constant $y_{0}$ from actual responses ${\bar{y}}_{ij}$, which are unsigned integers, before IPR calibration. A suitable value for $y_{0}$ is $\mathrm{round}(\bar{y})$, where $\bar{y}$ is the mean of ${\bar{y}}_{i}$. Such a subtraction reduces the range of power terms, *i.e.*, ${\left({\bar{y}}_{ij}-y_{0}\right)}^{k}$ replaces ${\bar{y}}_{ij}^{k}$, which in turn reduces the range of estimated parameters ${\hat{b}}_{jk}$. As shown (coloured red) in Figure 2, the same subtraction must be done prior to IPR correction. This refinement does not affect the floating-point SSE but it substantially reduces the fixed-point SSE for a given total wordlength.

## 4. Results and Discussion

This paper introduced polynomial-based methods and their fixed-point implementation for the calibration and correction of log CMOS image sensors. The theory involved provable mathematical deductions. Nevertheless, experimental results presented here illustrate how the theory is applied in practice. More importantly, the results also validate unproved assumptions of the theory.

In this section, floating-point calibration and fixed-point implementation results are given and discussed for the proposed methods and relevant literature methods. Correction results are also presented and compared for several approaches. In addition to these offline results, real-time correction results are given for a selected approach. All results, whether statistical values or actual images, use experimental data obtained at video rates with the log CMOS image sensor described in Section 2.1.

### 4.1. FPN and Photometric Calibration

**Figure 3:**
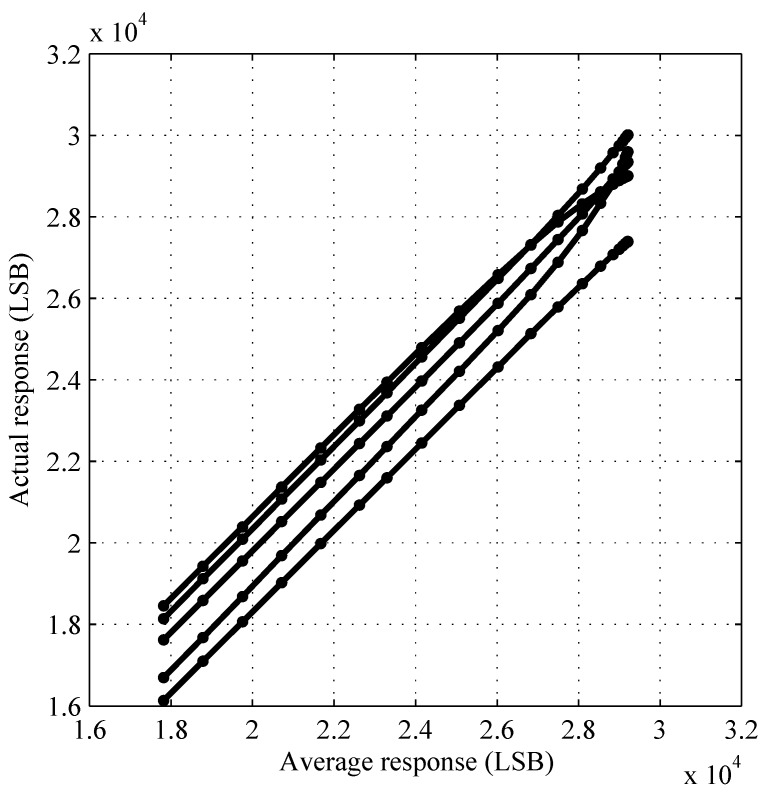
Actual response versus average response. Because of mismatch variation, which causes FPN, the actual response of any pixel—five are shown—varies relative to the average response of all pixels. The average response is considered to be an ideal response.

Figure 3 validates that actual responses are approximate linear functions of the average response, despite the highly nonlinear dependence of response on luminance, over a wide DR, as shown in Figure 1. In the absence of FPN, the actual response of any pixel equals the average response of all pixels.

The PR and IPR methods, also called PR(*p*) and IPR(*q*) methods in this section, use degree *p* and degree *q* polynomial models, respectively, having p+1 and q+1 parameters per pixel (ppp). Considering Figure 3 as an example, the PR method models actual responses as polynomial functions of average responses, whereas the IPR method does the opposite. One of the reasons the latter proves more useful is because the purpose of FPN correction, when divorced from photometric correction, is to take non-ideal, *i.e.*, actual, responses as inputs and give ideal, e.g., average, responses as outputs.

An FPN, or relative, calibration involves the estimation of parameters, per pixel, for an imaging model that captures pixel-to-pixel variations. A photometric, or absolute, calibration involves the estimation of parameters, per imager, for an imaging model that captures imager-to-imager variations. Although joint FPN-photometric calibration and correction is possible, as discussed below for literature methods, the common practice with linear imagers is to separate them. In this paper, a separate approach is likewise taken for monotonic nonlinear imagers, in particular for a log CMOS image sensor.

Otim *et al.* [14] have presented three related models, founded upon circuit analysis, which could be used for joint FPN-photometric calibration and correction of log CMOS image sensors. For these models, an equivalent algebraic representation, more suitable for this discussion, is as follows:

(50) $${\bar{y}}_{ij}=\left\{\begin{array}{ll} a_{j}+b_{j}\ln\left(x_{i}\right)+\epsilon_{ij}\text{,} & c_{j}\ll x_{i}\ll d_{j}\\ a_{j}+b_{j}\ln\left(c_{j}+x_{i}\right)+\epsilon_{ij}\text{,} & c_{j}+x_{i}\ll d_{j}\\ a_{j}^{\prime }+b_{j}^{\prime }\ln\left(\exp\left(\sqrt{\frac{c_{j}+x_{i}}{d_{j}}}\right)-1\right)+\epsilon_{ij}\text{,} & \text{otherwise} \end{array}\right.$$

In Equation (50), the first two cases, called the offset-gain (OG) and offset-gain-bias (OGB) models, follow from earlier work by Joseph and Collins [10]. We name the third case the offset-gain-bias-knee (OGBK) model. In the OGBK model, $c_{j}$ and $d_{j}$ are luminances at which the response function bends due to dark current and strong inversion effects, respectively. When luminances of interest are not bright enough for the strong inversion effects, the model simplifies to the OGB model. Dark current effects may also be ignored, resulting in the OG model. Parameters $a_{j}$ (or $a_{j}^{\prime }$), $b_{j}$ (or $b_{j}^{\prime }$), $c_{j}$, and $d_{j}$ are called the offset, gain, bias, and knee, respectively. Two are functions of the others, as follows:

(51) $$\begin{array}{cc} a_{j} & =a_{j}^{\prime }-\left(b_{j}^{\prime }/2\right)\ln\left(d_{j}\right) \end{array}$$

(52) $$\begin{array}{cc} b_{j} & =b_{j}^{\prime }/2 \end{array}$$

Section 2 explained the PR and IPR calibrations, both of which entail general linear regression. Calibration of the OGB and OGBK models requires nonlinear regression. Linearized regression may be used with the OG model, by taking $\ln(x_{i})$ as the independent variable. For each method, the overall RMS residual FPN, ${\hat{\sigma }}^{d}$, and the RMS residual FPN per luminance, ${\hat{\sigma }}_{i}^{d}$, are computed as follows:

(53) $$\begin{array}{cc} {\hat{\sigma }}^{d} & =\sqrt{\frac{1}{(m-l)n}\sum_{i=1}^{m}\sum_{j=1}^{n}{\left(r_{ij}^{d}\right)}^{2}} \end{array}$$

(54) $$\begin{array}{cc} {\hat{\sigma }}_{i}^{d} & =\sqrt{\frac{m}{(m-l)n}\sum_{j=1}^{n}{\left(r_{ij}^{d}\right)}^{2}} \end{array}$$

where complexities, *l*, and residual FPN, $r_{ij}^{d}$, are given in Table 1. As residual FPN is a form of spatial distortion in corrected images, a superscript d is used, in contrast to n for temporal noise.

**Table 1:** Summary of FPN calibration methods. For each method, the complexity is the number of parameters per pixel (ppp) required for FPN correction, and the residual FPN is the (weighted) error, at each luminance $x_{i}$ and pixel *j*, of the fitted response.

Method	Complexity (*l*)	Residual FPN ($r_{\mathrm{ij}}^{d}$)
PR(*p*)	p+1	${\bar{y}}_{ij}-\left({\bar{y}}_{i}+\sum_{k=0}^{p}{\hat{a}}_{jk}{\bar{y}}_{i}^{k}\right)$
IPR(*q*)	q+1	${\hat{w}}_{ij}\left({\bar{y}}_{i}-\left({\bar{y}}_{ij}+\sum_{k=0}^{q}{\hat{b}}_{jk}{\bar{y}}_{ij}^{k}\right)\right)$
OG	2	${\bar{y}}_{ij}-\left({\hat{a}}_{j}+{\hat{b}}_{j}\ln\left(x_{i}\right)\right)$
OGB	3	${\bar{y}}_{ij}-\left({\hat{a}}_{j}+{\hat{b}}_{j}\ln\left({\hat{c}}_{j}+x_{i}\right)\right)$
OGBK	4	${\bar{y}}_{ij}-({\hat{a}}_{j}^{\prime }+{\hat{b}}_{j}^{\prime }\ln\left(\exp\left(\sqrt{\left({\hat{c}}_{j}+x_{i}\right)/{\hat{d}}_{j}}\right)-1\right)$

**Figure 4:**
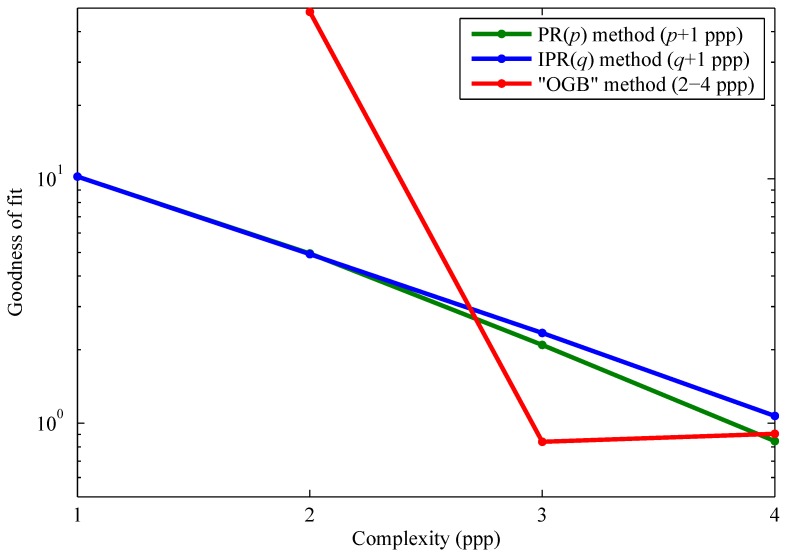
Comparison of FPN calibration methods. The overall goodness of fit is the ratio of overall RMS residual FPN to overall RMS temporal noise. This paper introduced the polynomial regression (PR) and inverse polynomial regression (IPR) methods, whereas the offset-gain-bias (OGB) and related methods are taken from the literature.

Figure 4 gives the overall goodness of fit, defined as ${\hat{\sigma }}^{d}/{\hat{\sigma }}^{n}$, for all calibration methods summarized in Table 1. When this ratio is less than or equal to about one (or $10^{0}$), it means that FPN is effectively calibrated. Thus, FPN is effectively calibrated by the PR(3), IPR(3), OGB, and OGBK methods.

The overall goodness of fit of the PR and IPR calibrations are comparable for quadratic and cubic polynomial models, as shown in Figure 4. However, with the PR method, it would be difficult and very difficult to perform FPN correction using quadratic and cubic polynomials, respectively, because all roots must be computed, for each pixel, and the correct root must be selected, also for each pixel. Thus, the IPR method is much preferred for these polynomial degrees. Note that overall goodnesses of fit are identical for lower degrees, *i.e.*, p=q=0 and p=q=1. This is expected mathematically.

**Figure 5:**
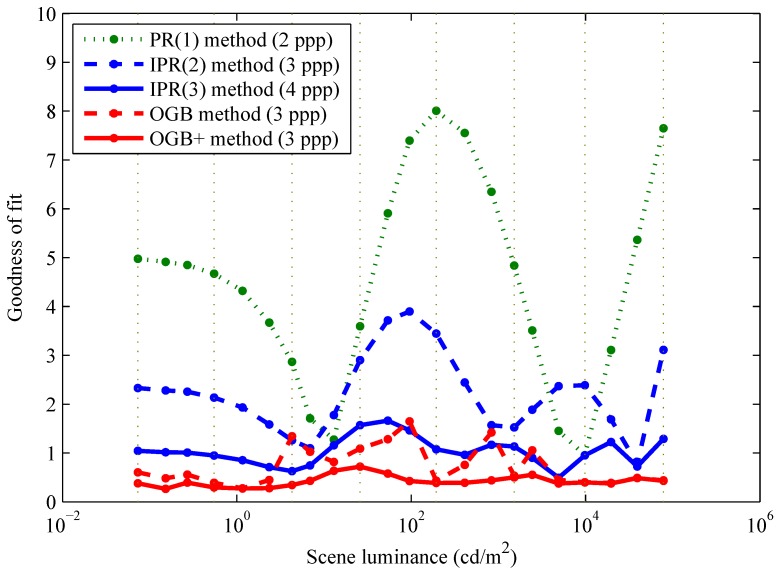
Comparing goodness of fit per luminance. This ratio divides RMS residual FPN per luminance by overall RMS temporal noise. Unlike the OGB method, the OGB+ method, also taken from the literature, corrects for luminance errors. However, unlike both OGB methods, the IPR method requires only arithmetic operations for FPN correction.

Figure 5 shows the goodness of fit per luminance, defined as ${\hat{\sigma }}_{i}^{d}/{\hat{\sigma }}^{n}$, versus luminance, $x_{i}$, for the linear PR, quadratic IPR, cubic IPR, OGB, and OGB+ (see below) calibrations. As shown in Figure 4, the OGBK method, at the cost of increased complexity, provided no benefit over the OGB method.

In theory, a good FPN calibration should be equally good at all luminances of interest. In practice, OGB+ method aside, all calibrations exhibit dependence of goodness on luminance, which is worst for the linear PR and quadratic IPR calibrations. The cubic IPR and OGB calibrations exhibit goodnesses that are relatively independent of luminance. For both calibrations, goodnesses are approximately one, which means residual FPN is comparable to temporal noise at each luminance over the wide DR.

Luminance dependence of the OGB method, which entails a joint FPN-photometric calibration, may be attributed to measurement errors in luminances $x_{i}$. The OGB+ method [10] is a complex approach, used for our prior publication [3], that factors these out. As explained in Section 1, the objective of this paper, as with other published papers, is to simplify correction even at some expense of accuracy. The IPR(3) method is almost as accurate as the OGB method but requires only arithmetic operations.

FPN calibration using the IPR method enables mapping of the actual response of each pixel (ordinate in Figure 3) to an ideal response (abscissa in Figure 3). Photometric calibration using the ISI method enables mapping of this ideal response (ordinate in Figure 1) to scene luminance (abscissa in Figure 1). ISI calibration is done by constructing a cubic Hermite spline, which guarantees monotonicity, to map from $\bar{y}$ to lnx using the 22 data points $\left({\bar{y}}_{i},\ln x_{i}\right)$ shown in Figure 1. The result, called an inverse spline because it represents an inverse function, is plotted in Figure 1 (solid cyan line).

### 4.2. Fixed-Point Implementation

Whereas calibration is done once for an image sensor, FPN and photometric correction must be done repeatedly and in real time for a video-rate image sensor. Although a floating-point implementation is feasible for small pixel arrays operating at low frame rates, a fixed-point implementation is expected to be more scalable, especially for low-power applications. Another advantage of fixed point is reduced storage requirements, relative to floating point, for the parameters estimated during calibration.

A fixed-point implementation, however, may cause a loss of performance. For FPN calibration, this is quantifiable by comparing to the overall goodness of fit, ${\hat{\sigma }}^{d}/{\hat{\sigma }}^{n}$, introduced in the previous section. The fixed-point version, ${\hat{\sigma }}^{D}/{\hat{\sigma }}^{n}$, uses a revised overall RMS residual FPN, ${\hat{\sigma }}^{D}$, defined as follows:

(55) $$\begin{array}{cc} {\hat{\sigma }}^{D} & =\sqrt{\frac{1}{(m-l)n}\sum_{i=1}^{m}\sum_{j=1}^{n}{\hat{w}}_{ij}^{2}{\left({\bar{y}}_{i}-{\hat{Y}}_{ij}\right)}^{2}} \end{array}$$

where corrected images ${\hat{Y}}_{ij}$ are obtained from raw images ${\bar{y}}_{ij}$ according to Figure 2, with $y_{j}$ and ${\hat{Y}}_{j}$ replaced by ${\bar{y}}_{ij}$ and ${\hat{Y}}_{ij}$, respectively. As before, the complexity, *l*, of this IPR(*q*) calibration is q+1.

**Figure 6:**
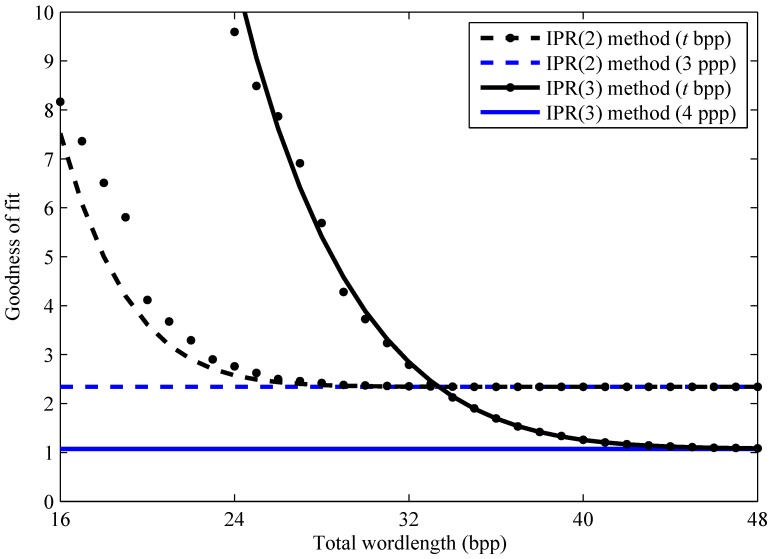
Fixed and floating-point implementations. For the fixed-point FPN calibration, actual (dots) and modeled (curved lines) goodness results are shown. Both are computed, at each total wordlength *t*, after optimization of the model. The floating-point FPN calibration results (horizontal lines) are the limiting values. Here, bpp stands for bits per pixel.

Figure 6 plots the overall goodnesses of fit, for fixed-point implementations of the IPR(2) and IPR(3) calibrations, versus the total wordlength, *t*, used to quantize the estimated parameters, ${\hat{b}}_{jk}$. In addition to the actual goodnesses, as defined in the previous paragraph, modeled goodnesses are shown. The only difference is in the calculation of ${\hat{Y}}_{ij}$ in Equation (55). As explained in Section 3, an approximate but differentiable model is defined, which is optimized to minimize the impact of parameter quantization.

Figure 6 demonstrates that the fixed-point results converge on the floating-point ones—the horizontal lines—with a sufficient total wordlength, *t*. Moreover, Figure 6 validates the differentiable model as an approximation of the actual fixed-point results. This is important because the model is used to determine how many bits, $t_{k}$, to allocate to each parameter, as well as their binary-point positions, $s_{k}$. Examples are given in Table 2. IPR parameters ${\hat{b}}_{jk}$ are quantized to integers ${\hat{B}}_{jk}$, according to Equation (45).

**Table 2:** Details of fixed-point implementations. Total wordlengths *t*, in bpp, are selected to be integer multiples of whole bytes. Here, $t_{k}$ represents the wordlengths, also in bpp, of parameters ${\hat{B}}_{jk}$, shown in Figure 2, and $s_{k}$ represents their binary-point positions. For each pixel *j*, floating-point parameters ${\hat{b}}_{jk}$ are quantized to get integer parameters ${\hat{B}}_{jk}$.

	(a) IPR(2) method (tbpp)						(b) IPR(3) method (tbpp)							
t	$t_{0}$	$t_{1}$	$t_{2}$	$s_{0}$	$s_{1}$	$s_{2}$	$t_{0}$	$t_{1}$	$t_{2}$	$t_{3}$	$s_{0}$	$s_{1}$	$s_{2}$	$s_{3}$
16	7	6	3	8	−4	−15	4	4	5	3	10	−1	−14	−26
24	10	8	6	5	−6	−18	6	7	6	5	8	−4	−16	−28
32	12	11	9	3	−9	−21	8	9	8	7	6	−6	−18	−30
40	15	14	11	0	−12	−23	10	11	10	9	4	−8	−20	−32
48	18	16	14	−3	−14	−26	12	13	12	11	2	−10	−22	−34

Figure 6 also illustrates that fixed-point considerations may be the deciding factor when choosing between polynomial degrees. For example, if one decided to limit the FPN correction parameters to four or fewer bytes per pixel, *i.e.*, t≤32, then there is no advantage in using cubic over quadratic polynomials. Considering that an integer number of bytes or nibbles (half bytes) tends to be efficient from a hardware perspective, there is little advantage in using a fixed-point IPR method for 28<t<36, at least with this particular image sensor. Accordingly, one could use a fixed-point IPR(2) method with three bytes per pixel (24bpp) or a fixed-point IPR(3) method with five bytes per pixel (40bpp).

Hoefflinger [13] reports a fixed-point implementation for a log CMOS image sensor with an active pixel sensor (APS) architecture. His implementation uses 24bpp to represent parameters of the OGB model. While he achieves a residual FPN comparable to temporal noise, the temporal noise, relative to signal, was 3.5 times (11dB) higher than with our log CMOS image sensor, based on a DPS array. The PSNR of his image sensor was 35dB [13], whereas the PSNR of ours is 46dB [3]. Furthermore, Hoefflinger does not show that his 24bpp fixed-point implementation is optimal in any sense.

A fixed-point implementation is also required for our photometric correction. As shown in Figure 2, this can be done simply using an LUT. The input and output of the FPN correction (*i.e.*, $y_{j}$ and ${\hat{Y}}_{j}$ in Figure 2, respectively) are both 16-bit integers. As such, an LUT with $2^{16}$ words, at most, may be pre-computed to perform ISI correction in real time. This correction is essentially an inverse mapping of the average response function shown in Figure 1. ISI correction may be effectively combined with “tone mapping,” explained in the next section. This specifies the size of each word in the LUT to be one byte. Thus, a 64 kilobyte LUT, at most, suffices to implement both photometric correction and tone mapping. Only one LUT is required to perform these operations for the whole pixel array.

### 4.3. FPN and Photometric Correction

Tone mapping refers to the processing used to properly display images from wide DR cameras [13]. Standard displays, such as monitors and printers, can depict a relatively narrow DR of intensities. For the purposes of this paper, which is not about tone mapping, a simple approach is adopted based on the sRGB specification [15], which is the default colour space of modern displays. Because our image sensor is monochromatic, the colour processing part of the sRGB specification is ignored.

For an image with *n* pixels, let ${\hat{x}}_{j}$ be the estimated scene luminance of the *j*th pixel, where 1≤j≤n. The displayed image $I_{j}$, which is an integer from 0 to 255 at each pixel, is computed as follows:

(56) $$\begin{array}{cc} I_{j} & =\left\{\begin{array}{cc} \mathrm{round}(255{\left({\hat{x}}_{j}/x_{0}\right)}^{1/2.2})\text{,} & {\hat{x}}_{j}<x_{0}\\ 255\text{,} & \text{otherwise} \end{array}\right. \end{array}$$

where saturation is given by the white point, $x_{0}$, and “gamma correction” by the exponent, 1/2.2.

According to the sRGB specification, modern displays simulate the gamma response of legacy cathode ray tubes (CRTs). This “CRT” gamma cancels out the exponent in Equation (56) to achieve overall a linear mapping from estimated luminances ${\hat{x}}_{j}$ to displayed tones. Given $\ln{\hat{x}}_{j}$ instead of ${\hat{x}}_{j}$, as with ISI correction, the above tone mapping may be rewritten as follows, where *ℓ* represents lnx:

(57) $$\begin{array}{cc} I_{j} & =\left\{\begin{array}{cc} \mathrm{round}(255\exp\left(\left({\hat{\ell }}_{j}-\ell_{0}\right)/2.2\right))\text{,} & {\hat{\ell }}_{j}<\ell_{0}\\ 255\text{,} & \text{otherwise} \end{array}\right. \end{array}$$

Median filtering is employed to remove salt-and-pepper noise caused by “dead” pixels, *i.e.*, outliers where responses are essentially useless. For interior pixels, the neighbourhood is a five-pixel cross made up of the pixel and its four nearest neighbours. For border and corner pixels, the pixel and its nearest two border pixels make up a three-pixel neighbourhood. These are the smallest symmetric neighbourhoods possible, where odd sizes ensure that means are never needed to compute medians.

The chosen median filter has low complexity, which is good for real-time processing. Furthermore, the avoidance of means implies that the median filter may be placed equally before or after any monotonic transform. ISI correction and simple tone mapping are each monotonic transforms. Although the median filter may be placed before either of these transforms, with no impact on final images, median filtering is most efficiently done after the simple tone mapping because of the 8bpp format.

As described in Section 2.1, additional images $y_{ij}$ were collected of the 22 calibration scenes, each of uniform luminance $x_{i}$, where 1≤i≤22. These images were not used for calibration. Also, unlike the calibration images ${\bar{y}}_{ij}$, these images were not averaged over time and, thus, include unfiltered temporal noise. For brevity, every third additional image, *i.e.*, where i=1,4,⋯22, was selected. Corresponding luminances, which cover a DR of 121dB, are indicated by vertical lines in Figure 1 and Figure 5.

**Figure 7:**
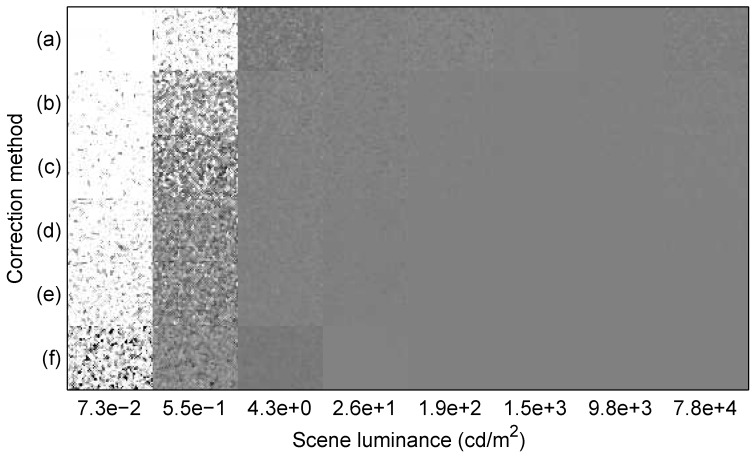
Corrected images versus correction method. Top to bottom: (**a**) the PR(1) method (2ppp); (**b**) the IPR(2) method (24bpp); (**c**) the IPR(2) method (3ppp); (**d**) the IPR(3) method (40bpp); (**e**) the IPR(3) method (4ppp); and (**f**) the OGB method (3ppp). With perfect FPN and photometric correction, all pixels would have a uniform grey value.

Figure 7 depicts the outcome of FPN correction, photometric correction, simple tone mapping, and median filtering for the eight selected additional images, using six different correction methods, including fixed-point implementations of two IPR methods. At each selected luminance $x_{i}$, which ranged from $7.3\times 10^{-2}$ to $7.8\times 10^{4}\;\mathrm{cd}/m^{2}$, the white point chosen for the simple tone mapping was ${\left(255/128\right)}^{2.2}x_{i}$. With this choice, perfect FPN and photometric correction would result in a uniform mid-level grey image, *i.e.*, $I_{j}$ would equal 128LSB for all pixels, after simple tone mapping.

To interpret Figure 7, consider both Figure 1 and Figure 5. A better goodness per luminance, *i.e.*, a lower value, in Figure 5 means that residual FPN is less significant relative to temporal noise. However, a more horizontal slope in Figure 1, for the average response as a function of log luminance, means that residual FPN and temporal noise have a greater impact on estimated luminance and, hence, image quality.

Figure 7 depicts the results of floating-point PR(1), IPR(2), and IPR(3), plus ISI, corrections. In these cases, Equation (57) specifies the tone mapping. Image quality improves with increasing polynomial degree, especially going from the linear to the quadratic model. Note the non-uniform greyness with the PR(1) results, even at higher luminances. The figure also depicts the results of fixed-point IPR(2) and IPR(3) corrections, using 24 and 40bpp, respectively. In these cases, Equation (57) specifies the tone mapping with ${\hat{L}}_{j}$ instead of ${\hat{\ell }}_{j}$, where ${\hat{L}}_{j}$ is the result of the fixed-point ISI correction, as shown in Figure 2. Compared to the corresponding floating-point results, there is little to no difference.

Figure 7 also depicts the results of the floating-point OGB method, for which Equation (56) specifies the tone mapping. Although not ideal, image quality is better at $7.3\times 10^{-2}\;\mathrm{cd}/m^{2}$, compared to all other methods. However, image quality is worse at $4.3\;\mathrm{cd}/m^{2}$. What is happening is a problem with the photometric correction, not with the FPN correction, because the OGB method is sensitive to measurement errors in $x_{i}$, which determines the white point. Overall, compared to the OGB method, the quadratic and cubic IPR, plus ISI, methods offer satisfactory image quality, over a wide DR, and this performance is achievable using a fixed-point implementation based largely on simple arithmetic.

**Figure 8:**
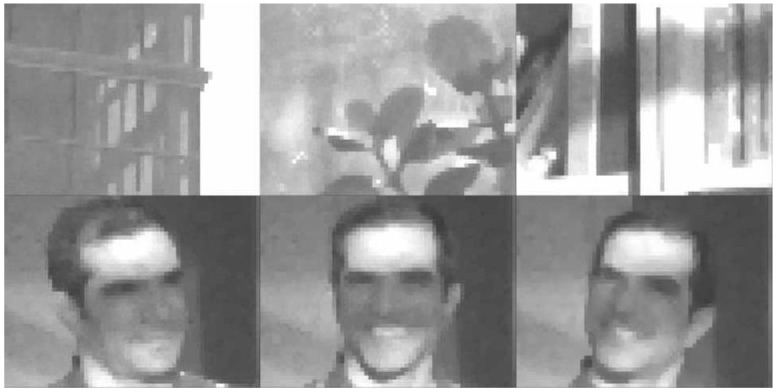
Polynomial-based correction in real time. Because the visual quality of quadratic FPN correction was deemed sufficient, with simple tone mapping, and the fixed-point work was unfinished at the time, the IPR(2) method (3ppp) was implemented at a 30Hz frame rate. Top row (left to right): a building against the sky; a plant by a window; and a bookshelf in sunlight. Bottom row: three views of a face with highlights and shadows.

The IPR(2) and ISI methods were programmed to operate in real time on a desktop computer that controlled our image sensor. Videos were displayed and recorded of multiple non-uniform scenes. Each used a different white point chosen manually. Figure 8 depicts several frames taken from these videos. Notwithstanding the low spatial resolution of the camera, which had only 48×64 pixels [3], the image quality is good after tone mapping, further validation for the polynomial-based methods.

## 5. Conclusions

This paper proposed novel methods, based on low-degree polynomials, for FPN and photometric correction. Although developed for log CMOS image sensors, which achieve wide DRs easily at video rates, the proposed methods are not tied to any circuit model, unlike previous work. They may be applied to any image sensor, provided pixel responses are monotonic with respect to light stimulus.

When FPN calibration and correction are done using the proposed IPR method, correction may be implemented solely with arithmetic operations. Photometric calibration and correction are done using the proposed ISI method, where correction may be implemented solely with logic and arithmetic operations. Computational complexity may be further reduced using a proposed fixed-point implementation, which introduces bit shifting and an LUT to the FPN and photometric correction, respectively. To minimize the number of bits required for FPN correction, a Lagrangian function is defined and optimized.

The theory was validated using a log CMOS image sensor, operating at video rates, having relatively low temporal noise, thanks to in-pixel ADCs. With IPR correction, the residual FPN was made as low as the temporal noise. Together with ISI correction, the approach proved comparable, over a wide DR, to a leading approach from the literature that performs a joint FPN-photometric correction. Equivalent results were achieved using a fixed-point implementation. In conclusion, the proposed method achieved satisfactory performance, over a wide DR, but with low computational complexity.
